# Qualitative assessment of women’s satisfaction with maternal health care in referral hospitals in Nigeria

**DOI:** 10.1186/s12978-017-0305-6

**Published:** 2017-03-16

**Authors:** Friday Okonofua, Rosemary Ogu, Kingsley Agholor, Ola Okike, Rukiyat Abdus-salam, Mohammed Gana, Abdullahi Randawa, Eghe Abe, Adetoye Durodola, Hadiza Galadanci, The WHARC WHO FMOH MNCH Implementation Research Study Team, Eze Nwokocha, Lorretta Ntoimo, Oye Ekiti

**Affiliations:** 1 Women’s Health and Action Research Centre WHARC, Benin, Edo State Nigeria; 2 University of Medical Sciences, Ondo, Ondo State Nigeria; 3 University of Port Harcourt, Port Harcourt, Rivers State Nigeria; 4 Central Hospital, Warri, Delta State Nigeria; 5 Karshi General Hospital, Federal Capital Territory, Abuja, Nigeria; 6 Adeoyo Maternity Hospital, Ibadan, Oyo State Nigeria; 7 General Hospital, Minna, Niger State Nigeria; 8 Ahmadu Bello University Teaching Hospital, Zaria, Kaduna State Nigeria; 9 Central Hospital, Benin, Edo State Nigeria; 10 General Hospital, Ijaye, Abeokuta, Ogun State Nigeria; 11 Aminu Kano Teaching Hospital, Kano, Kano State Nigeria

**Keywords:** Maternal Healthcare, Maternal and child health, Comprehensive obstetric care, Delays, Nigerian women, Referral hospitals, Focus groups discussion, Respectful care in childbirth

## Abstract

**Background:**

Available evidence suggests that the low use of antenatal, delivery, and post-natal services by Nigerian women may be due to their perceptions of low quality of care in health facilities. This study investigated the perceptions of women regarding their satisfaction with the maternity services offered in secondary and tertiary hospitals in Nigeria.

**Methods:**

Five focus group discussions (FGDs) were held with women in eight secondary and tertiary hospitals in four of the six geo-political zones of the country. In all, 40 FGDs were held with women attending antenatal and post-natal clinics in the hospitals. The questions assessed women’s level of satisfaction with the care they received in the hospitals, their views on what needed to be done to improve patients’ satisfaction, and the overall quality of maternity services in the hospitals. The discussions were audio-taped, transcribed, and analyzed by themes using Atlas ti computer software.

**Results:**

Few of the participants expressed satisfaction with the quality of care they received during antenatal, intrapartum, and postnatal care. Many had areas of dissatisfaction, or were not satisfied at all with the quality of care. Reasons for dissatisfaction included poor staff attitude, long waiting time, poor attention to women in labour, high cost of services, and sub-standard facilities. These sources of dissatisfaction were given as the reasons why women often preferred traditional rather than modern facility based maternity care. The recommendations they made for improving maternity care were also consistent with their perceptions of the gaps and inadequacies. These included the improvement of hospital facilities, re-organization of services to eliminate delays, the training and re-training of health workers, and feedback/counseling and education of women.

**Conclusion:**

A women-friendly approach to delivery of maternal health care based on adequate response to women’s concerns and experiences of health care will be critical to curbing women’s dissatisfaction with modern facility based health care, improving access to maternal health, and reducing maternal morbidity and mortality in Nigeria.

**Trial registration:**

Trial Registration Number NCTR No: 91540209. Nigeria Clinical Trials Registry. http://www.nctr.nhrec.net/. Registered April 14th 2016.

## Plain english summary

The use of unskilled providers by women for antenatal and delivery care is a major cause of maternal mortality in sub-Saharan Africa. One reason why women do not use hospitals for care during pregnancy is their low level of satisfaction with hospital services. This study investigated women’s experiences in maternity hospitals and assessed their level of satisfaction with the services provided.

### Methods

Women who attended maternity care in each of eight hospitals in Nigeria were organized into five discussion groups. In all, the study obtained the views of 40 groups of women in four out of the six geo-political zones in Nigeria.

### Results

Most participants reported dissatisfaction with the quality of care they received during pregnancy, delivery and after delivery. Many had specific areas of dissatisfaction, or were dissatisfied with the overall quality of care. Reasons for dissatisfaction included poor staff attitudes, long waiting times, poor attention to women in labour, and substandard facilities. t = They also stated that these were the reasons why many women used traditional birth attendants rather than modern hospitals for childbirth care. In order to change the situation, the women recommended that hospital facilities be improved, delays in hospitals should be avoided, and that feedback/counseling/education of women should be provided.

### Conclusion

A women-friendly approach to delivery of maternal healthcare based on adequate responses to women’s concerns is necessary to improve women’s access to maternal health and to reduce the number of women harmed or who die during childbirth.

## Background

Low demand for modern, facility-based maternal health care is a major public health concern in Nigeria. Recent reports from the Nigeria Demographic and Health Survey [1] indicate that less than 65% of pregnant women in Nigeria use modern health-facilities for antenatal services; fewer than 35% receive skilled birth attendance at delivery; while fewer than 65% seek postnatal services. It has also been estimated that fewer than a third of women who experience pregnancy complications have access to some form of emergency obstetric services [2], the quality of which may not be optimal [3]. By contrast, many women prefer to receive antenatal, delivery, and post-natal care either at home or in the homes of traditional and faith-based birth attendants [4, 5]. There is now some evidence indicating that the use of unskilled providers for antenatal and delivery care is one of the major predisposing factors to high rates of maternal morbidity and mortality in many sub-Saharan African countries [6].

Several reasons have been proffered for this pattern of poor utilization of maternal health services by Nigerian women. These include inability to pay for services [7], cultural preferences [8], and attitudes of health providers [9, 10]. However, negligible attention has been paid to the perceptions of women who use health facilities on their satisfaction with the quality of maternity care. Yet, it is one indicator that can be worked on swiftly to improve the quality of maternity care and to increase the demand by patients for such services [11].

The primary aim of this study was to investigate women’s level of satisfaction with the quality of care in the three dimensions of structure, process and outcomes in the continuum of maternity care - antenatal, intrapatum, and postnatal services. Structure included physical environment, cleanliness, availability of adequate human resources, and medicines and supplies. Process included interpersonal behavior of providers, privacy, promptness of care, cognitive care (prenatal counseling and health education), perception of provider’s competency, and preference for providers. Outcome is the health and survival status of the mother [12–14]. A secondary aim of the study was to identify, based on recommendations by the women, cost-effective measures that could be put in place to improve women-friendly approaches for the management of obstetric services in secondary and tertiary health institutions in Nigeria. We believe that this framework will improve the utilization of obstetric services by women eventually leading to the reduction of maternal and neonatal morbidity and mortality in the country.

## Methods

**Fig 1:**
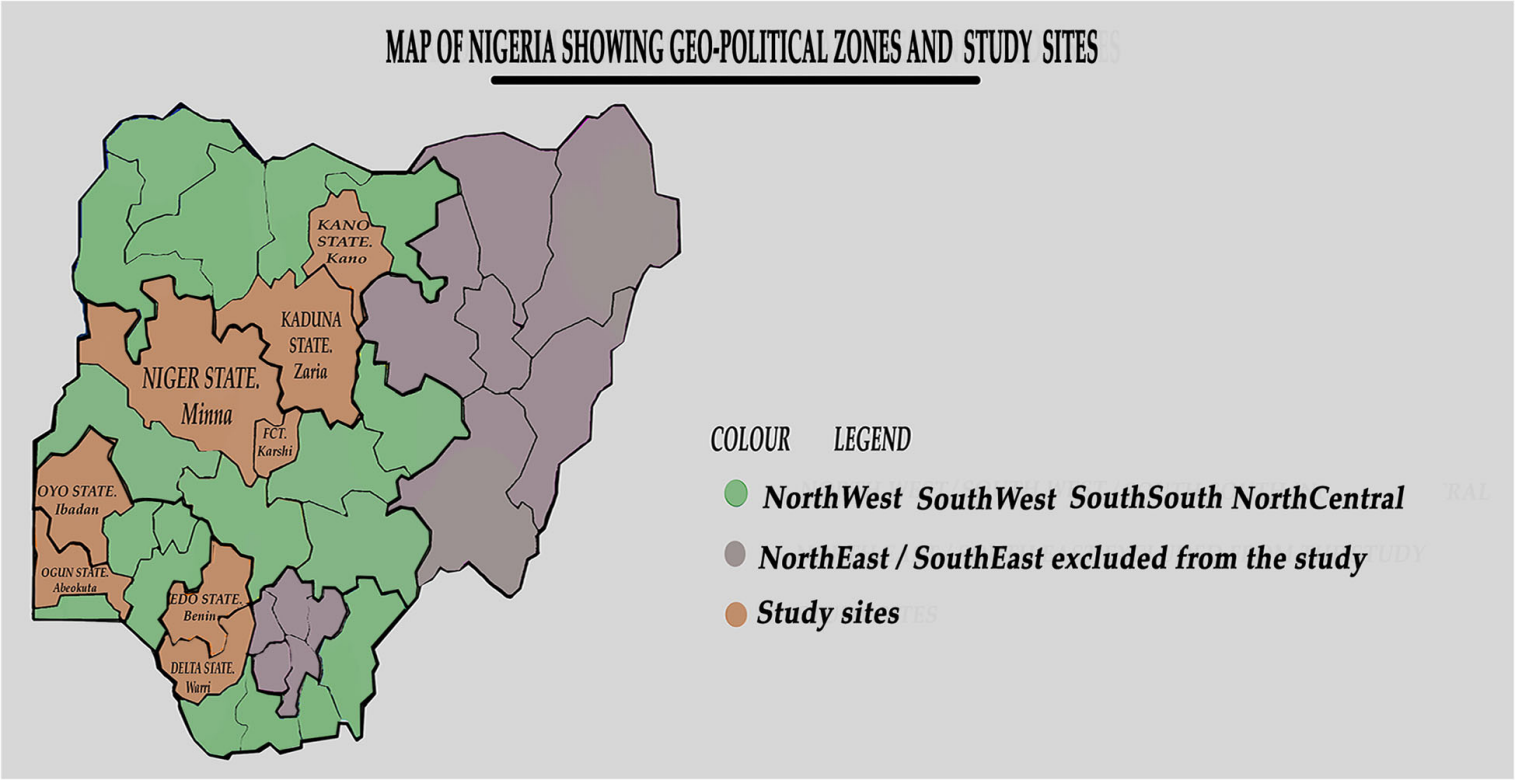
Map of Nigeria showing geo-political zones and the study sites

Focus Group Discussions (FGDs) were conducted with women attending maternity clinics in eight tertiary and secondary hospitals in Nigeria to determine their perceptions about the care they received. The hospitals were purposively selected from four out of six geo-political zones in Nigeria (see Fig. 1). Administratively, Nigeria is made up of thirty-six states and a Federal Capital Territoty (FCT), Abuja, which are grouped into six geo-political zones: Northcentral, Northeast, Northwest, Southeast, South-south and Southwest. Two hospitals were selected from each zone except the Northeast and Southeast. The Northeast was not included because of the current insurgency by a terrorist group in that zone, thus the eastern zone in the South was also left out of the study. In the Northwest, the Aminu Kano Teaching Hospital in Kano, Kano State and the Ahmadu Bello University Teaching Hospital, Zaria in Kaduna State were selected. In the Northcentral, General Hospital, Minna, Niger State and Karshi General Hospital, FCT, Abuja were slected. Two facilities were selected from the Southwest: Adeoyo Maternity Hospital, Ibadan, Oyo State; State Hospital, Ijaye, Abeokuta, Ogun state; and Central Hospital, Warri, Delta State and General Hospital, Benin City, Edo State were from the South-south.

Five FGDs were conducted in each health facility in 2015. Each FGD consisted of 8 to 12 pregnant women or those who had recently delivered that were attending antenatal or postnatal clinics in the hospital. The participants were recruited through personal contacts when they came for antenatal and postnatal clinics. Two FGDs per hospital consisted of women attending antenatal care, while three FGDs per hospital consisted of women attending postnatal care. The FGD guide was developed and revised by the team leaders at a central meeting held in the project coordinating office. The guide was pre-tested in that location and again pre-tested in the individual study sites before application. In particular, the study guides were translated into the local languages appropriate for the study sites and used for women groups not literate in English.

Trained researchers facilitated the FGDs., asking questions, about the women’s perceptions of the quality of care received in the hospital. They were asked about their levels of satisfaction and to make recommendations on what needed to be done to improve the quality of services in the hospitals. All FGDs were audio-taped and transcribed in each hospital. Transcripts in local languages were back-translated to English before final analysis. The transcripts were then forwarded to the coordinating Centre where they were analysed qualitatively.

### Data analysis

Analysis was conducted in the Department of Sociology and Anthropology at the University of Ibadan, Nigeria. Qualitative data analysis package Atlas ti 6.2 was used for coding. At the first step, transcripts were assigned into Atlas ti and open coding was used to generate themes following the FGD guide for the study, and emerging concepts. At the second level, the codes were organized into analytical categories in form of code families in relation to the study objectives. Data analysis consisted of description of the content and form of transcripts conducted in each site, followed by a review and comparison of the results between the sites. The results enabled us to gain insights into the nature of the policies in each site as well as women’s perceptions and level of satisfaction regarding the quality of services.

### Ethical approval

Ethical approval for the study was obtained from the World Health Organization and the National Health Research Ethics Committee (NHREC) of Nigeria – number NHREC/01/01/2007 – 16/07/2014, renewed in 2015 with NHREC 01/01/20047-12/12/2015b.

## Results

### Women’s opinions on quality of maternity care

Three categories of responses emerged from the women’s narratives on satisfaction with the quality of care: satisfied with all the services; satisfied, but not with all the services,; and not satisfied. Diverse reasons were given for their stated satisfaction and dissatisfaction, and many women freely proffered their perspectives on what could be done to improve the quality of maternity care in health facilities.

### Satisfaction with quality of care

The results on satisfaction are presented according to the themes that emerged from the women’s narratives and the reasons for their perspectives.

#### Satisfied with all services

Few of the participants from the Southwest and Northwest expressed satisfaction with all the services they received during antenatal, intrapartum, and postnatal care. For example, a participant from the Southwest said: “The quality of care given in this hospital is very good and perfect. This is the hospital I attended in my first pregnancy” (FGD 3, Oyo). Inquiring into what informed their satisfaction, some participants gave reasons that revolved around structure and process, such as availability and ease of procurement of routine drugs, quality of antenatal services, good interpersonal relationship with many of the providers, positive attitudes by the nurses, adequate number of qualified doctors and nurses on ground, timely initiation, and good care. Commenting on the quality of care in the hospital she uses, a participant said: “they attend to us on time, and a nurse cannot give a patient drip except the doctor” (FGD 2, Kano). One participant gave a unique reason from the others, that her satisfaction is based on her husband’s preference. She said: “My husband prefers Adeoyo Maternity Hospital. This is because he believes one will receive specialist care from the doctors and benefit from the experience of the doctors” (FGD 2, Oyo). Although satisfied, some of the participants were specific about different types of care, such as antenatal and postnatal. A participant in FGD 2, Kaduna was of the opinion that “Patients access their services satisfactorily especially the patients who attend routine antenatal care services”. In another state, a discussant stated: “The quality of the care in this hospital is very good when it comes to antenatal and postnatal care” (FGD 1, Oyo).

#### Satisfied, but not in all services

Many women who said they were satisfied with the quality of care in the hospitals they attended also expressed dissatisfaction with some aspects of care. Verbal abuse and unfriendly attitude of the providers was an area of dissatisfaction expressed by the women. Commenting on this, a respondent said “they take care of people but they like “fighting” [abusing] people (FGD 5, Kano). Attempting to quantify her perception, a respondent opined: “the quality of care can be described as about 70% if I exclude their verbal abuse and attitude (FGD 5, Oyo). Narrating her experience with regard to verbal abuse, another participant said:

The doctors are friendly, as you can see one now is playing with me but the nurses, oh my Jesus Christ, whenever I talk, they will insult me. They will ask whether they are the ones who sent me here, and I pay for the services with my money; it is my own money that I will pay. They will ask me if they were the ones who sent me here. Many times I will be crying here, shouting here, no attention; they will be insulting me (FGD 2, Edo).

In FGD 1, Abuja, all the participants said the services were satisfactory, but nurses needed to make more effort to improve on their attitude to mothers. Other aspects of dissatisfaction expressed by women who were not totally satisfied with care, included stock outs, long waiting times, shortage of staff, dirty environment, non-compliance with health insurance, and the doctors’ industrial strike. On long waiting times, a respondent reported that “Maternity care Ade-Oyo Maternity Hospital is not time conscious, we have to get to the hospital as early as 5 am and this does not guarantee being attended to on that day” (FGD 2, Oyo). Speaking on the doctors’ strike, one of them said: “they should put an end to strike, now I came to see the doctor, they said they are on strike” (FGD 1, Kano).

#### Dissatisfied

Many of the respondents expressed outright dissatisfaction with the quality of maternity care they received in the health facilities. In one of the hospitals in Edo, when the question on satisfaction was asked, all the participants hissed and chorused: “they lack so many things; it’s not supposed to be like that” (FGD 3, Edo). Reasons for dissatisfaction included lack of and/or insufficient equipment, irregular electricity and water supply, inadequate number of doctors and other health care providers, long waiting time to retrieve folders and receive treatment, unfriendly attitudes of providers and other support staff, poor radiological and laboratory services, poor attention to women in labour, and late arrival to work by providers. Expressing her dissatisfaction, one woman said: “they tell you to come in the morning, you will sit and get tired and they won’t attend to you. I come here and get tired and still won’t see the doctor” (FGD 3, Kano). In this same FGD, another participant spoke of her experience: “in the scanning and x-ray unit, you can book for up to a month or two, they won’t refer you somewhere else to do it, and they won’t release the result promptly. In addition, she stressed that those in the lab should improve” (FGD 3, Kano). Speaking on the unfriendly attitude of providers and support staff, a participant said: “there are people in the record room at the antenatal clinic; they talk to people anyhow and even shout at people that are older than them” (FGD 4, Kano). Narrating experiences in the labour room, a participant averred: “they do not give enough attention to women in labour, some women will be shouting and crying and they still will not attend to them” (FGD 5, Kano). In Edo, a participant said:

What I know is that they don’t have good manner of approach, they always like to harass persons here. If you ask questions, it is just like a big sin. When you are asking questions you need to know your left and right. I don’t know the way they are acting here. I don’t understand! Even since yesterday night I was here telling them to come and do the test, they didn’t come, they were telling me they will come, they will come, but the pain is increasing. Because of the pain, I did not sleep well yesterday even to this afternoon the same thing, for me to eat now is a problem, I only take something liquid, how will I get well!. (FGD 2, Edo)

Asked to describe the quality of care in the hospital she attends, a participant responded: “I feel the quality of care offered in this facility is below average, when asked her reasons, she said: “The facility lacks medical doctors, particularly to manage emergency cases” (FGD 5, Kaduna).

However, some of the participants who were not satisfied with the quality of maternity care they received commended some aspects of services in their specific hospitals. One of them submitted that “procurement of drugs is made easy” (FGD 4, Kaduna). Another woman in the same group commented that “initiation of treatment is prompt at 8.00 am or.8.30 am”.

### Recommendations for improving obstetric care

As a follow-up question on the participants’ perception of the quality of maternity care, they were asked what aspects of obstetric care they think needed to be improved in the hospitals. In response, four thematic areas emerged in the recommendations. These include improvement of hospitals’ facilities, better organization of hospitals’ operations, staff training/development and re-orientation, and patient education.

#### Improvement of hospital facilities

Participants made extensive recommendations for improving the hospital facilities as these were generally reported to be in a poor state. Specific recommendations made included: expanding antenatal clinics and labour wards, providing hospital equipment and facilities to enable health workers do their jobs, providing more seats for patients waiting to see the doctor, increasing the number of hospitals and hospital beds, keeping toilets clean, and regular provision of water and electricity. One participant said: “There should be enough chairs and sheds for patients to sit at antenatal clinics. We sometimes sit on fences and doctors’ cars” (FGD 1, Oyo). Many participants from the various hospitals spoke about the provision of adequate and comfortable seats for women who seek antenatal care. On facilities, another participant avowed: “because of the breakdown of machines in the laboratory, they will send us out for the test. The scan facility is poor in this hospital; in some private scan facilities, if you lie down you will be watching yourself, you will see all that they are doing but here it’s not like that” (FGD 5, Abuja). A typical opinion about the toilets and environments was in FGD 4, Edo, where a participant said: “environment, everywhere here is smelling, see, people are here now, toilet, everywhere is smelling, they should take care of the environment”.

#### Better organization of hospitals’ operations

Participants were most vehement on the need to improve the operations of the hospitals to make them more action-oriented and to remove delays. These include the adoption of the appointment system (whereby patients are given time to attend the clinics) so that patients do not have to wait for prolonged periods before they see health workers, reduction in the numbers of unnecessary episiotomies and cesarean sections, better organization of clinical work, reduction in costs of hospital services, and making drugs available so that patients do not have to go out in the night to buy needed drugs. A participant opined: “the practice of buying drugs in the middle of the night evokes a sense of fear of being haunted by the spirits” (FGD 3, Kaduna). Submitting her views on waiting time, a participant said: “appointment system should be adopted in antenatal clinics so that patients can be attended to on first-come-first-served basis” (FGD 3, Oyo). Another participant recommended that: “measures should be put in place to address the problem of delay during consultations” (FGD 2, Kaduna). Expressing her view on reducing waiting time, another participant opined: “the nurses should be separated, those for antenatal and for others so that the results can come out on time” (FGD 3, Kano). A group in Kano recommended that HIV tests should not be made compulsory in maternity units, as this requirement often drives women away from using the facilities. One of the participants stated: “The tests they do – they should remove the HIV test, it scares people” (FGD 1, Kano).

#### Staff training/development and re-orientation

Regarding staff development, there was a general consensus among the FGD participants that there is a need to increase the number of staff working in various sections of the maternity ward, as existing staff are often over-worked. Staff also need to be re-trained to make them more friendly to patients, more prompt and responsive in their dealings with patients, and more service oriented. Increasing the number of providers was a recommendation that cut across all the FGDs. A participant in Kano said: “they should increase the number of workers” (FGD 5). In Kaduna, a woman avowed: “more qualified and experienced health personnel should be appointed to manage all conditions relating to obstetrics” (FGD 5). Another participant in Oyo said: “the number of health care providers should be increased” (FGD 4). Expressing her opinion with specific reference to the labour room, a participant said “they should increase the number of staff in the labour room and stop maltreating people” (FGD 2, Kano). On providers’ unfriendly attitude, a participant reported: “There is need to improve inter-personal contact with the patients. Patients need reassurance and counseling” (FGD 2, Kaduna). Another woman stated: “health workers should be more caring” (FGD 3, Ogun).

#### Patient’s education

A key recommendation was the need to encourage women to share positive experiences in hospitals rather than negative experiences so as not to scare women away from the hospital. Some groups also recommended the enlightenment of women on the importance of seeking early care in pregnancy. Speaking on this, a participant said: “Pregnant women should be adequately enlightened on the benefits of attending the ABUTH, Shika for their obstetric care services without minding the cost of treatment” (FGD 1, Kaduna).

## Discussion

From women’s perspectives, pregnant women experience three categories of challenges when they use public maternity (maternal health) care services. These include: inherent lack of information which prevents women from seeking early care, difficulties they experience during transportation to hospital, and inadequacies in the health care delivery system [5, 8, 11]. Of these, deficiencies in the maternal health care delivery system featured most prominently in this assessment, for which participating women provided profuse explanations and examples. The women were dissatisfied with the quality of care they received during antenatal, intrapartum, and postnatal care. Poor staff attitude, long waiting time, poor attention to women in labour, high cost of services and inadequate facilities were the major reasons for dissatisfaction.

The results of the study indicate that women in all geo-political zones of the country are very knowledgeable about the problem of maternal mortality and about the medical and social problems that lead to death. To our surprise, women mentioned specific medical causes of maternal mortality with utmost accuracy and indeed, there was no evidence in all of the focus groups of wrong use of medical terms and terminology by women. It was also of interest that the socio-economic factors that lead to maternal mortality that were elucidated by the women also tallied very well with those repeatedly mentioned in the scientific literature and by programmers and policymakers across the country.

Arising from this elucidation of challenges, women also made recommendations on ways to address the situation. The recommendations included the improvement and expansion of hospital facilities, better organization of clinical services to reduce delays and mis-management, the training and re-orientation of health workers and the education/counseling of women. Indeed, this review of challenges and the recommendations for rectification are consistent with previous recommendations [5, 7–9], and with the outcomes of the composite research carried out as part of this assessment. For example, our careful and objective assessment of time spent by women to obtain services in the hospitals indicate that women sometimes spend as much as four to five hours to obtain specific services [15]. This is aside from the time they spend in getting to the health facilities, which when calculated in economic terms amount to huge losses not only to the women and their families but to the entire country. Delays are also characteristic of poor quality service, which results in poor outcomes for mothers and their babies. Surely, the application of the appointment system recommended by women in this study, and which is now the norm in many high performing maternity centres around the world [16] is the best way out of this problem. Hospitals should also identify reduction of delays as one of the women-friendly approaches to provision of care, which should be periodically evaluated and appropriately rewarded.

Poor attitude of staff also featured prominently in this feedback provided by women with the recommendation made for staff training, re-training and re-orientation. Poor attitude of staff may be due to heavy work-load and limited incentives. Strategic approaches for building the right numbers and quality of staff to match the population of women seeking maternity care would be useful. Additionally, the results of a recent study [3] reported that health workers in South-west Nigeria lack adequate knowledge about the principles and practice of modern maternity care.. Thus, poor attitude of staff reported by women in this study may be a manifestation of the inherent poor knowledge of clinical procedures and ancillary practices by health workers. It would therefore be of utmost importance to focus on staff training and re-training on obstetric care and its implementation methods as a way to overcome this problem. In this regard, building the capacity of staff to use standard operational clinical guidelines and protocols will be helpful.

Patients’ education was also cited as an important recommendation made by women for the re-organization of maternal health services. In particular, the recommendation for women’s education to enable them focus on reporting positive outcomes to other women was particularly insightful. This was mentioned by some of the FGDs as a way to move women away from using traditional methods to using facility-based maternity care. This is consistent with the social network theory [17], which posits that positive or negative information can be widely disseminated through social networks, especially if such information impacts the social lives of individuals significantly. However, it has to be noted that women can only report positive results if those results actually happened. Therefore, the improvement of facilities and health workers’ attitudes will greatly help the process of re-orienting women towards positive reporting of their experiences. Additionally, patients’ education will help women manage their clinical conditions and should include training on compliance and supportive self-care.

Only a few studies have objectively investigated women’s perceptions of the quality of maternity services in Nigeria. Extant literature revealed a few studies in Nigeria based on single maternity units that address women’s concerns about maternal health care services [18–20]. The major strength of this study is its nation-wide focus with women in four geo-political zones and from different socio-cultural backgrounds being provided the opportunity to offer their views in a value-free manner. Additionally, the fact that women perceived this to be an externally driven project, not influenced by stakeholders within the individual hospitals enabled them to speak freely and without inhibition.

A unique question asked in this study that has been little investigated in previous studies was why women used traditional birth attendants and home care rather than modern, facility-based maternal health services. Contrary to previous studies, which suggested that women inherently prefer traditional forms of care [4, 5], the results of this study indicate that preference for traditional care may be accentuated by the failure of modern health facility care to address the needs of women. It is evident therefore that correcting the deficiencies identified in hospitals by women, would be one way to increase the use of skilled birth attendants (in facilities) needed to reduce maternal morbidity and mortality. Specific information to women coupled with incentives may also help to shift women away from traditional birth attendants to modern, facility-based maternal health care.. The major limitation of the study was our inability to cover the six geo-political zones of the country, but instead confined the study to four zones. This was because of the high rate of insurgency in the North-east zone at the time of the study, which made it impossible for secondary and tertiary hospitals to function in that zone. To balance the deficit, we also decide to exclude the corresponding South-east zone in the southern part of the country. However, due to the socio-cultural similarities between the three southern zones and also between the three northern zones, we believe the non-representation of these two zones will not substantially reduce the depth and quality of the findings. Our inclusion of women who were still using the services in the hospitals rather than those who had completed treatment helped to reduce recall bias, and does not attenuate the information they provided on their experiences with service delivery in the hospitals.

## Conclusion

In conclusion, the results of this study indicate that women have differing levels of dissatisfaction with maternity services offered by public maternity hospitals in Nigeria. Their many recommendations made for improvement suggests the need for the strategic repositioning of secondary and tertiary hospitals to become more “women friendly”. A women-friendly approach to delivery of maternal health care based on adequate response to women’s concerns and experiences of health care will be critical to curbing women’s dissatisfaction with modern facility based health care; improving access to maternal health, and reducing maternal morbidity and mortality in Nigeria.
